# How subjective CT image quality assessment becomes surprisingly reliable: pairwise comparisons instead of Likert scale

**DOI:** 10.1007/s00330-023-10493-7

**Published:** 2024-01-02

**Authors:** Eva J. I. Hoeijmakers, Bibi Martens, Babs M. F. Hendriks, Casper Mihl, Razvan L. Miclea, Walter H. Backes, Joachim E. Wildberger, Frank M. Zijta, Hester A. Gietema, Patricia J. Nelemans, Cécile R. L. P. N. Jeukens

**Affiliations:** 1 Department of Radiology and Nuclear Medicine, Maastricht University Medical Centre+, P. Debyelaan 25, Maastricht, 6229 HX The Netherlands; 2 CARIM School for Cardiovascular Diseases, Maastricht University, Universiteitssingel 50, Maastricht, 6229 ER The Netherlands; 3 Department of Neurology and School for Mental health and Neuroscience (MheNs), Maastricht University Medical Centre+, P. Debyelaan 25, Maastricht, 6229 HX The Netherlands; 4 GROW School for Oncology and Reproduction, Maastricht University, Universiteitssingel 50, Maastricht, 6229 ER The Netherlands; 5 Department of Epidemiology, Maastricht University, Universiteitssingel 50, Maastricht, 6229 ER The Netherlands

**Keywords:** Computed tomography (X-ray), Interobserver variability, Intraobserver variability

## Abstract

**Objectives:**

The aim of this study is to improve the reliability of subjective IQ assessment using a pairwise comparison (PC) method instead of a Likert scale method in abdominal CT scans.

**Methods:**

Abdominal CT scans (single-center) were retrospectively selected between September 2019 and February 2020 in a prior study. Sample variance in IQ was obtained by adding artificial noise using dedicated reconstruction software, including reconstructions with filtered backprojection and varying iterative reconstruction strengths. Two datasets (each *n* = 50) were composed with either higher or lower IQ variation with the 25 original scans being part of both datasets. Using in-house developed software, six observers (five radiologists, one resident) rated both datasets via both the PC method (forcing observers to choose preferred scans out of pairs of scans resulting in a ranking) and a 5-point Likert scale. The PC method was optimized using a sorting algorithm to minimize necessary comparisons. The inter- and intraobserver agreements were assessed for both methods with the intraclass correlation coefficient (ICC).

**Results:**

Twenty-five patients (mean age 61 years ± 15.5; 56% men) were evaluated. The ICC for interobserver agreement for the high-variation dataset increased from 0.665 (95%CI 0.396–0.814) to 0.785 (95%CI 0.676–0.867) when the PC method was used instead of a Likert scale. For the low-variation dataset, the ICC increased from 0.276 (95%CI 0.034–0.500) to 0.562 (95%CI 0.337–0.729). Intraobserver agreement increased for four out of six observers.

**Conclusion:**

The PC method is more reliable for subjective IQ assessment indicated by improved inter- and intraobserver agreement.

**Clinical relevance statement:**

This study shows that the pairwise comparison method is a more reliable method for subjective image quality assessment. Improved reliability is of key importance for optimization studies, validation of automatic image quality assessment algorithms, and training of AI algorithms.

**Key Points:**

*• Subjective assessment of diagnostic image quality via Likert scale has limited reliability.*

*• A pairwise comparison method improves the inter- and intraobserver agreement.*

*• The pairwise comparison method is more reliable for CT optimization studies.*

**Graphical Abstract:**

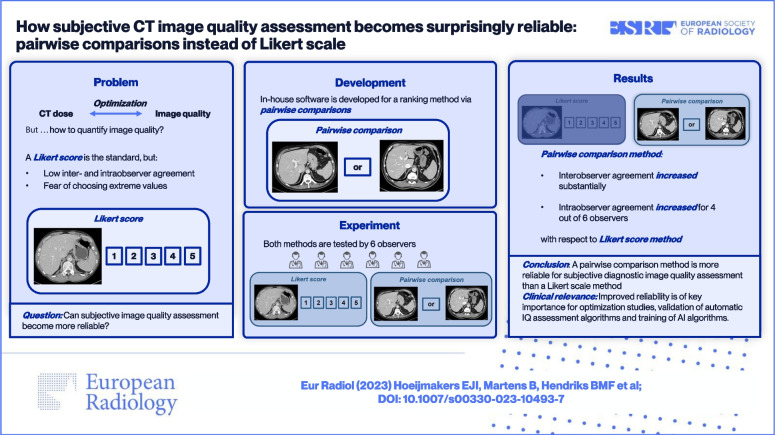

## Introduction

Computed tomography (CT) is one of the most important diagnostic imaging tools in daily clinical practice which requires sufficient image quality (IQ). However, increasing IQ, by decreasing image noise, implicates a higher radiation dose, which may lead to an increased lifetime attributable risk of cancer [1]. Optimization studies try to find the balance between radiation dose and IQ. However, reliable assessment of IQ remains a challenge [2, 3].

IQ is frequently assessed subjectively using a fixed (typically 5-point) Likert scale [4]. Subjective assessments can provide comprehensive information about IQ, as it takes human perception into account. However, it is prone to variability because of different interpretations of the points on the scale [5–7]. IQ is also commonly evaluated with objective parameters such as signal-to-noise ratio and contrast-to-noise ratio in specific regions of interest. Although these parameters are quantifiable, they may not always accurately reflect the radiologist’s perception of diagnostic IQ [8]. More advanced automated algorithms may be preferable for assessing IQ because of their objectivity and they do not require human observer time [9–11]. However, the development of automated algorithms requires validation using subjective IQ assessment, which could benefit from a more reliable method compared to the standard Likert scale method.

In a pairwise comparison (PC) method, the observer is repeatedly forced to choose the preferred scan out of pairs until all scans are ranked [12]. Previous studies in other disciplines suggest that the Likert scale has a low inter- and intraobserver agreement, while a PC method may be more accurate and sensitive even for small differences within the scans [5, 13–16]. When using the Likert scale method, the observers are often trained on the dataset or provided with reference scans to give an absolute score. However, studies suggest that this does not provide significant added value [13, 14]. In the PC method, the scans are rated in reference to each other avoiding the need for training or additional reference scans as well [12–17]. Additionally, observers tend to be hesitant to select the extreme Likert scores out of fear of a better or worse scan showing up afterwards [16]. This results in a bias to the middle of the scale and provides an evaluation with a less refined classification than the PC method.

This study aims to show that the reliability of subjectively scoring IQ improves by using the PC method instead of the Likert scale method in abdominal CT examinations.

## Material and methods

A waiver of written informed consent was provided by the local ethical committee and institutional review board, as retrospective data were analyzed anonymously (ref METC 2017-0250).

### Study design

Data collected for a prior publication is used [18], consisting of abdominal CT scans from 30 unique patients that were retrospectively selected between September 2019 and February 2020. All scans had a reference tube voltage and tube current of respectively 120 kV_ref_ and 150 mAs_ref_, with a slice collimation of 192 × 0.6 mm and gantry rotation time of 0.5 s. Scans were acquired at 90 kV with a dosing factor of 0.4 g I/kg contrast media and reconstructed with iterative reconstruction (IR) level 2. Movement artifacts were applied as exclusion criteria. To obtain more variation in the IQ of the scans, noise was inserted artificially using proprietary dedicated CT reconstruction software (version 13.0.0.1, prototype software, Siemens Healthineers, information on validation of the software can be found in [18]) by simulating scans at 60, 70, 80, and 90% of the original (100%) tube current. Additionally, the simulated scans were reconstructed using filtered backprojection (FBP) and IR levels 2, 3, and 4. This resulted in 510 CT scans (the original scans and 16 additional reconstructions per patient).

To investigate the reliability of subjective assessment on respectively more and less evident IQ differences, two datasets both containing 50 scans were constructed out of the total database by (1) including the original scans and (2) choosing scans obtained with a higher and lower variation in reconstruction methods and tube current values. The original scans were part of both datasets and each dataset was complemented by randomly selecting scans (not more than one per patient) from the total database such that the desired composition was achieved.

### PC method and Likert scale method

A Python-based software program was in-house developed for IQ scoring using the PC method and the Likert scale method, featuring a user interface designed to closely mimic diagnostic viewing systems and operate similarly, e.g., possibility to scroll through the slices and to adjust the window settings.

The PC method repeatedly presented the observers with pairs of CT scans and forced them to select the scan with the highest IQ, continuing until all scans could be ranked. As comparing all possible pairs of 50 scans requires 1225 comparisons, the Ford-Johnson algorithm was applied in the software which reduced the required number of comparisons to approximately 220 [13, 19]. This sorting algorithm assumes the transitivity relation of the scans: if scan A is preferred over B, and B is preferred over C, then preference of A over C is implied, thus eliminating the need to assess the latter. Depending on the previous preference, the algorithm generated the next pair presented to the observer; thus, the pairs that needed to be scored were unique for every observer and assessment. The PC method resulted in a ranking of the scans for each observer (low to high IQ).

For the Likert scale method, scans were presented one by one in random order and observers were prompted to click on the corresponding Likert score for overall IQ (1, very poor; 2, poor; 3, moderate; 4, good; 5, very good) [4].

### Image quality assessment

Five radiologists (C.M., F.Z., H.G., B.M., R.M.) and one radiology resident (B.H.) with respectively 12-, 12-, 10-, 7-, 13-, and 4 years of experience participated in the study. IQ was rated by the observers on diagnostic screens while being blinded to scan and patient information. Standard window level settings (window level = 40 HU; window width = 400 HU) for abdominal CT examinations were applied, but observers were allowed to adjust them. The observers were instructed to assess the scans based on overall diagnostic IQ. All observers rated the first dataset using both the PC method and the Likert scale method, and then, after at least a 2- week time interval, they rated the second dataset using the two methods. The original scans present in both datasets were used to evaluate the intraobserver agreement. Both assessment methods were performed without prior training or a reference image.

### Data analysis

Both the inter- and intraobserver agreements were quantified using the intraclass correlation coefficient (ICC). The ICC is a reliability parameter that ranges from 0 to 1, with 1 indicating total agreement between observers [20], calculated with statistical software (SPSS, v29.0; IBM Corp). For the calculation of the ICC, the two-way random method was used for absolute agreement. The average-measures ICCs were presented for interobserver agreement, representing the reliability of the average values of multiple observers. For intraobserver agreement, the single-measures ICCs were presented, representing the reliability of one single observer.

Graphical methods were used to visualize the interobserver agreement in ranking by different observers when using the PC method. Scans were sorted by the median of the ranking scores from all six observers. Per scan, the range of rankings assigned by the observers is visualized via boxplots where a smaller boxplot indicates more agreement. Colors are added to the boxplots indicating the median Likert score received by the observers. For the ICCs of the intraobserver agreement, the original scans present in both datasets were used as they were rated twice. For each observer, the assigned ranks at repeated measurements were visualized in a graph with a line between the assigned ranks.

Confusion matrices were used to visualize the inter- and intraobserver agreement when using Likert scores. The Likert scores are indicated on the horizontal and vertical axes and each cell in the matrix represents the number of scans that received a particular combination of Likert scores as assigned by two observers or at repeated measurements. A diagonal matrix would indicate perfect agreement.

## Results

**Fig. 1:**
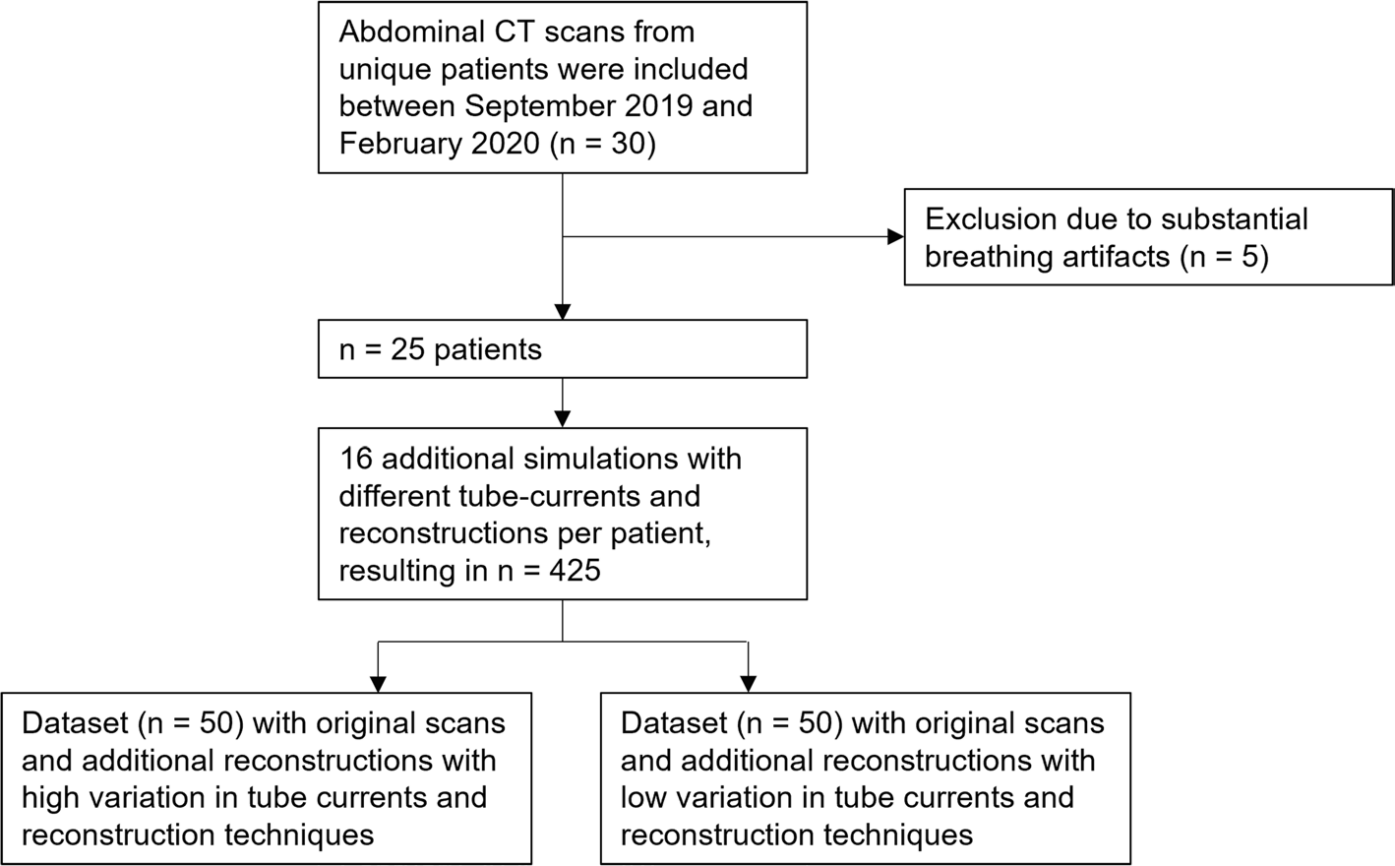
Flow diagram with selection of the high- and low-variation datasets

Data from five patients were excluded because of substantial breathing artifacts (Fig. 1). The baseline characteristics of the population are depicted in Table 1. The final compositions of the high and low IQ variation datasets are given in respectively Table 2a and b, with both datasets containing the 25 original scans and in addition 25 scans with either a high or low variety of tube currents and reconstruction techniques.

**Table 1:** Baseline characteristics

*Parameters*	*N=25*
Age (years)	61.1 ± 15.5
Sex (% male)	14 (56 %)
Body weight (kg)	72.6 ± 9.6
Height (m)	1.7 ± 0.1
BMI (kg/m^-2^)	24.4 ± 2.1

**Table 2:** Composition of the high-variation dataset (a) and the low-variation dataset (b). Both datasets include the 25 original scans complemented by 25 scans, for each patient one, with either a high or low variation in reconstruction levels (IR, iterative reconstruction level; FBP, filtered backprojection) and simulated tube current (60–90% of the original tube current)

(a) High-variation dataset	60%	70%	80%	90%	100%
FBP	2	1	2	2	
IR2	2	2	1	2	25
IR3	2	2	2	1	
IR4	1	1	1	1	
(b) Low-variation dataset	60%	70%	80%	90%	100%
FBP					
IR2					25
IR3					
IR4			13	12	

**Fig. 2:**
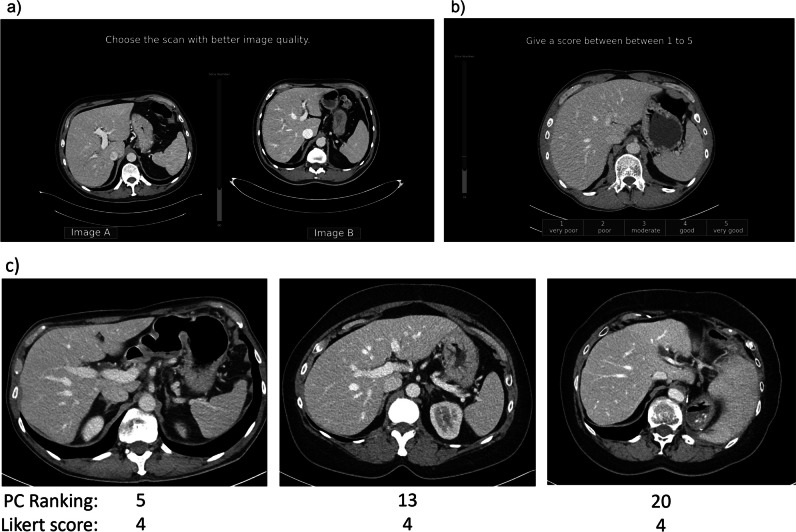
User interface of the in-house developed software for (**a**) the pairwise comparison method; **b** the Likert scale method. **c** Examples of 3 CT images ranked by an observer using the pairwise comparison method with corresponding Likert scores

The user interfaces of the in-house developed software are presented in Fig. 2a and b for the PC method and the Likert scale method respectively. Figure 2c shows the ranking of images by observer 5, while the same observer has assigned a Likert score of 4 to all three images.

### Interobserver agreement

The ICCs for both the PC method and the Likert scale method are presented in Table 3. The ICC of the high-variation dataset increased from 0.665 (95%CI 0.396–0.814) to 0.785 (95%CI 0.676–0.867) and the ICC of the low-variation dataset from 0.276 (95%CI 0.034–0.500) to 0.562 (95%CI 0.337–0.729) when using the PC method instead of the Likert scale method. Also, for both methods, the ICCs are higher for the high-variation dataset than for the low-variation dataset.

**Table 3:** Interobserver agreement. Intraclass correlation coefficients (ICC) for average values for both subjective scoring methods of image quality (pairwise comparison method and Likert scale) on the high- and low-variation dataset*s*

ICC	High-variation dataset	Low-variation dataset
Pairwise comparison method	0.785 (95%CI 0.676–0.867)	0.562 (95%CI 0.337–0.729)
Likert scale method	0.665 (95%CI 0.396–0.814)	0.276 (95%CI 0.034–0.500)

**Fig. 3:**
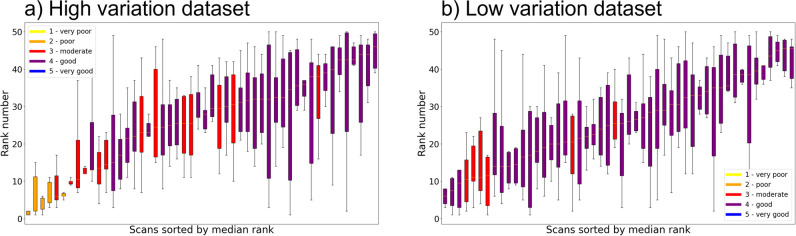
Boxplots representing the spread in ranks of the image quality assessment by all six observers using the pairwise comparison method. The boxes are sorted by the median of the six ranks received by the observers and colored by the median Likert score given by the observers. Results are given for the (**a**) high- and (**b**) low-variation datasets

Figure 3a and b present boxplots of the PC method results for the high- and low-variation datasets, respectively. Via the colors of the boxplots, it can be observed that a median Likert score of 3 or 4 was assigned to the majority of the scans (more than 90%). Ranking of the IQ according to the PC method shows some correlation with the Likert scores for IQ. For example, in Fig. 3a, 15 out of 18 scans that received a median Likert score of 3 or lower were ranked in the lowest half (left side of the figure) in the PC method and in Fig. 3b all six scans with 3 or lower were ranked in the lowest half.

**Fig. 4:**
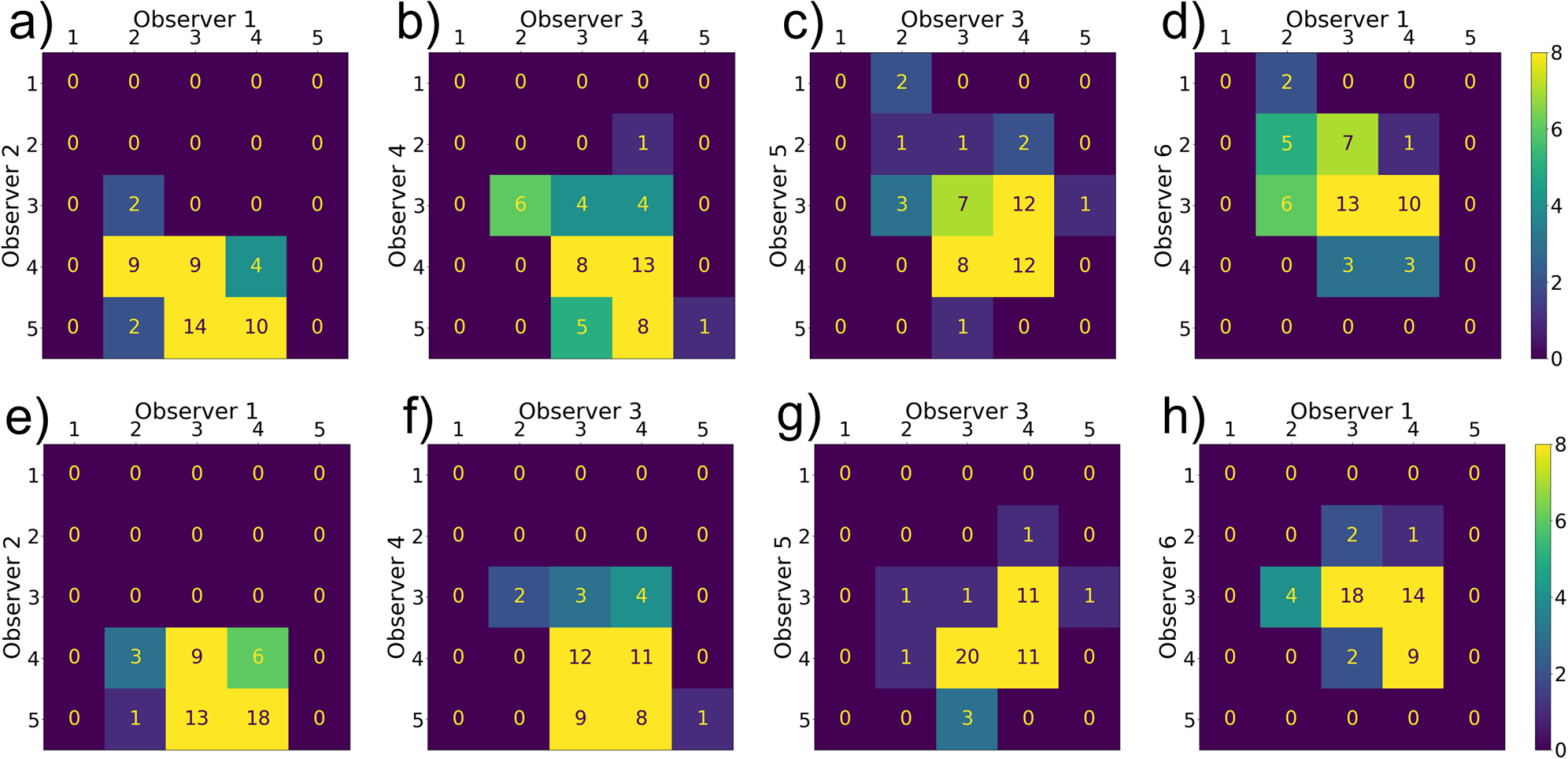
Confusion matrices of random combinations of two observers for the Likert scores of the high-variation dataset (**a**–**d**) and low-variation dataset (**e**–**h**). The horizontal and vertical axes give the Likert scores given by the observers. A diagonal matrix would indicate complete agreement

Figure 4 shows a representative selection of the confusion matrices for the Likert scale method for several pairs of observers for data from the high (Fig. 4a–d) and low (Fig. 4e–h) variation datasets. None of the matrices showed a clear diagonal which would have indicated high agreement. Frequently, an offset from the diagonal is observed, e.g., Fig. 4a, where observer 1 most of the time rates lower than observer 2. Also, some observers use the whole range of scores 1–5, e.g., observer 5 in Fig. 4c, while other observers mainly use Likert scores 3 and 4, e.g., observer 3. Sometimes large differences in scores are observed, in which one observer rates a scan with a score of 2 and another observer rates the same scan with a 5, e.g., Fig. 4e. No clear differences between the high-variation data and the low-variation data can be observed from the confusion matrices.

### Intraobserver agreement

Table 4 shows that the ICCs for intraobserver agreement increased substantially for four out of six observers when using the PC method instead of the Likert scale method. Observer 3 has approximately equal ICCs for both methods and for observer 4 the ICC decreased.

**Table 4:** Intraobserver agreement. Intraclass correlation coefficients (*ICC*) for both subjective scoring methods of image quality (pairwise comparison method and Likert scale) for repeated assessment by every observer

ICC	Observer 1	Observer 2	Observer 3	Observer 4	Observer 5	Observer 6
Pairwise comparison method	0.646 (95%CI 0.331–0.831)	0.321 (95%CI 0.000–0.640)	0.596 (95%CI 0.255–0.804)	0.078 (95%CI 0.000–0.468)	0.826 (95%CI 0.638–0.921)	0.705 (95%CI 0.425–0.862)
Likert scale method	0.097 (95%CI 0.000–0.450)	0.014 (95%CI 0.000–0.225)	0.610 (95%CI 0.288–0.809)	0.337 (95%CI −0.077 to 0.656)	0.267 (95%CI 0.000–0.583)	0.588 (95%CI 0.238–0.800)

**Fig. 5:**
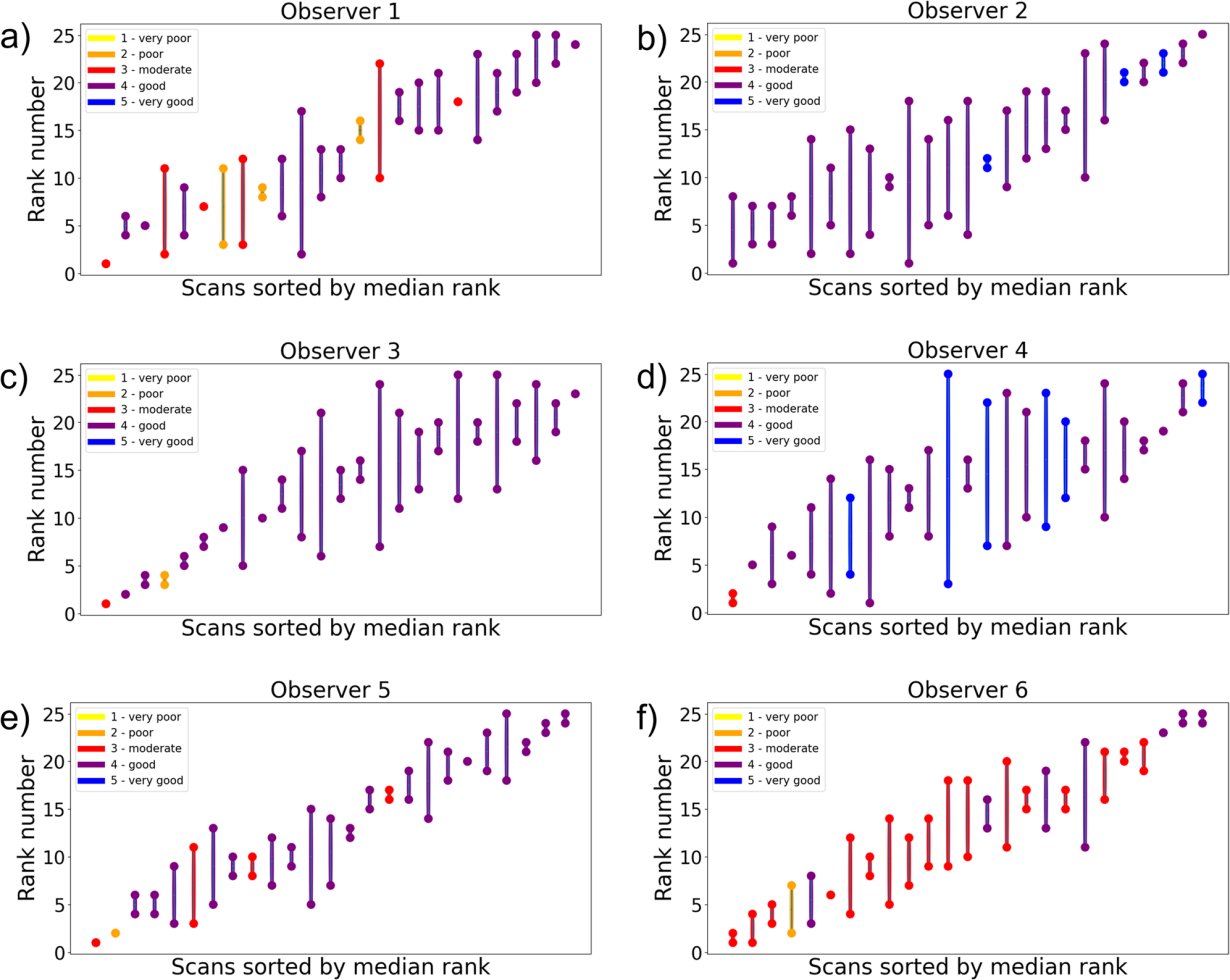
The agreement between two assessments using the pairwise comparison method for every single observer. For each scan, two dots are shown corresponding to the ranking in assessments 1 and 2. The color represents the mean Likert score given by the observer in the two assessments. The scans are sorted by the median rank. As an example, for observer 5, nearly every scan received a Likert score 4 (purple), despite the images consistently having distinct rankings in both assessments

Figure 5 shows the ranking results using the PC method at two measurement moments for each observer. It can be observed that the scans with extreme median ranks show less spread in the repeated assessment than the scans with less extreme median ranks, indicating more agreement on the extreme ranks. Furthermore, since the color represents the median Likert score from the two assessments for each observer, it is evident that most scans received a median Likert score of 3 or 4 independent of the observer, while demonstrating distinct rankings. The colors also show that some observers score consistently lower than other observers.

**Fig. 6:**
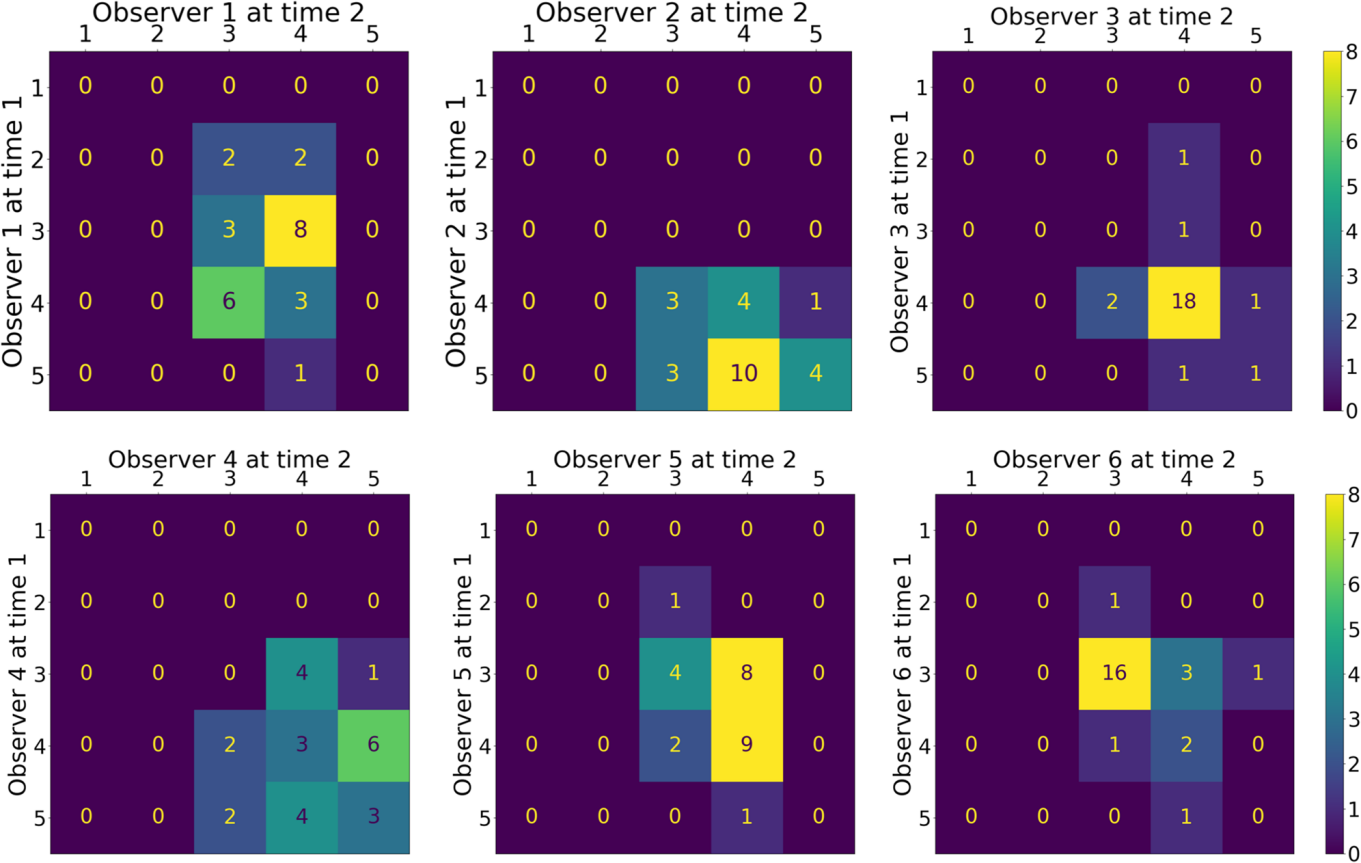
Confusion matrices of the repeated assessment for every single observer (intraobserver agreement). The horizontal (upper) and vertical axes give the Likert scores given by the observers. A diagonal matrix would indicate total agreement

The confusion matrices of the repeated assessment of IQ with the Likert scale method are shown in Fig. 6. It can be observed that observer 6 and observer 3 in particular had little variation in the Likert scores because the majority of the CT scans received the same score on IQ (64% and 72% respectively received a score of 4 during both assessments).

## Discussion

This study showed that the reliability of subjective IQ assessment improved by using the PC method instead of the standard Likert scale method in the evaluation of abdominal CT scans. When using the PC method, the ICCs for interobserver agreement increased substantially irrespective of the variation in IQ between scans and for the intraobserver agreement they increased substantially for four out of six observers.

Likert scale assessments are frequently performed using a reference standard or after receiving prior training, as one might argue that this is beneficial for the accuracy. However, literature indicates that offering a reference standard or prior training does not appear to yield substantial added value. Phelps et al [14] found that having observers repeat their Likert assessments and providing them with reference best and worst images did not noticeably improve accuracy. Similarly, Mantiuk et al [13] did not find any benefit from the addition of a reference scan to a Likert score method. For a fair comparison of both methods, it was decided to not use additional reference scans or prior training for either method.

Subjective evaluation of diagnostic IQ is a frequently used method to test hypotheses in diagnostic imaging and, generally, this is performed using a Likert scale method, e.g., in [6, 8, 10, 18, 21–27]. Mantiuk et al [13] compared the four most common assessment methods, including a PC method, on photo and video quality and found the PC method to be the most accurate and time-efficient. They also investigated the PC method with the addition of indicating how much the IQ differs on a continuous scale, but this did not improve the results. In medical IQ assessment, only a limited number of studies related to the PC method were reported. Phelps et al [14] compared the PC method to a Likert scale method on a set of 10 clinical chest radiographs with different degrees of image sharpness (digitally blurred). They evaluated the agreement of both methods with the actual sharpness and found that the PC method performed better than the Likert scale method. Furthermore, Ellman et al [28] used a PC method in their CT dose optimization study and found a high interobserver agreement. To the best of our knowledge, our study is the first that compares both methods for CT scans.

The current study was performed using abdominal CT scans, but we expect that results are generalizable to other anatomies and imaging modalities as improvement of IQ assessment using the PC method was shown in a non-medical setting [13] and chest radiographs [14]. Future studies could validate this observation by assessing the effectiveness of the PC method across different modalities. In the present study, the observers were given the general instruction to rate the scans based on overall diagnostic IQ, but both methods can also be used with a more specific diagnostic question, for example “rate the image quality for discerning focal liver pathology.” This may enhance the level of agreement among the observers, particularly for the PC method. While for the Likert scale method, independent of the specificity of a diagnostic question, the same issues will remain, e.g., the lack of reference material and the tendency to refrain from extreme scores, a specific diagnostic question might simplify the choice between scans when using the PC method as demonstrated by Ellman et al [28].

The PC method is limited by the need for a sorting algorithm that greatly reduces the number of comparisons to be evaluated because it is not feasible for observers to evaluate all possible pairs of scans. However, sorting algorithms are typically better suited to work with numerical or lexicographical data, and may not be as effective when applied to subjective data such as diagnostic IQ, in which cyclic relations can occur and there is no ground truth [5, 13]. Application of the PC method without a sorting algorithm would prevent errors due to transitivity assumptions, and would therefore lead to even more reliable results but requires a significant additional time investment.

Although execution time is substantially reduced by using a sorting algorithm, it took the observers about three times longer to complete the assessment with the PC method compared to the Likert scale method. Therefore, the trade-off between execution time and reliability should be carefully considered. In many cases, the benefits of a more reliable subjective assessment and more refined classification will outweigh the additional time investment.

It is important to note that the PC method captures relative preferences and rankings; thus, it cannot be employed for the absolute evaluation of CT scans. On the other hand, the Likert scale, despite being an ordinal scale, allows for the establishment of a predefined threshold for diagnostically sufficient image quality.

In conclusion, the findings of this study indicate that the use of the PC method improves the reliability of subjective IQ assessment of clinical CT scans compared to the Likert scale method, even for small differences in IQ. This may greatly benefit future optimization studies and research on automatically determined IQ parameters for which validation with subjectively perceived IQ is of importance. Additionally, a large dataset subjectively scored on IQ in a reliable manner might be useful as training data for machine learning algorithms on automatic IQ determination. When part of a quality assurance program, automatically determined IQ can be monitored continuously, making it possible to observe small changes in IQ at an early stage.

## Abbreviations

FBP: Filtered backprojection
ICC: Intraclass correlation coefficient
IQ: Image quality
IR: Iterative reconstruction
PC: Pairwise comparison

## Declarations

### Guarantor

The scientific guarantor of this publication is Cécile R.L.P.N. Jeukens.

### Statistics and biometry

One of the authors (Patricia J. Nelemans, Maastricht University) has significant statistical expertise.

### Study subjects or cohorts overlap

Some study subjects or cohorts have been previously reported in B. Martens et al, 2022 (https://doi.org/10.1371/journal.pone.0266194).

### Methodology

• retrospective

• observational

• performed at one institution
